# Determinants of Interpatient Variability in Treosulfan Pharmacokinetics in AML Patients Undergoing Autologous Stem Cell Transplantation

**DOI:** 10.3390/ijms25158215

**Published:** 2024-07-27

**Authors:** Selin G. Ayçiçek, Dilara Akhoundova, Ulrike Bacher, Michael Hayoz, Yolanda Aebi, Carlo R. Largiadèr, Thomas Pabst

**Affiliations:** 1 Department of Medical Oncology, Inselspital, University of Bern, CH-3010 Bern, Switzerland; selin.aycicek@students.unibe.ch (S.G.A.); dilara.akhoundovasanoyan@insel.ch (D.A.); 2 Department of Hematology, Inselspital, University of Bern, CH-3010 Bern, Switzerland; veraulrike.bacher@insel.ch; 3 Center of Laboratory Medicine (ZLM), Inselspital, University of Bern, CH-3010 Bern, Switzerland; michael.hayoz@insel.ch (M.H.); yolanda.aebi@insel.ch (Y.A.); carlo.largiader@insel.ch (C.R.L.); 4 Department of Clinical Chemistry, Inselspital, University of Bern, CH-3010 Bern, Switzerland

**Keywords:** acute myeloid leukemia (AML), treosulfan, pharmacokinetics, interpatient variability, exposure, high-dose chemotherapy (HDCT), autologous stem cell transplantation (ASCT), adverse events, clinical outcome

## Abstract

Limited data on treosulfan pharmacokinetics in adults, particularly regarding autologous stem cell transplantation (ASCT) in acute myeloid leukemia (AML), is available to date. Furthermore, correlations between treosulfan exposure, toxicity, and clinical outcome remain understudied. In this single-center retrospective study, we analyzed data from 55 AML patients who underwent HDCT with treosulfan (14 g/m^2^) and melphalan (140 mg/m^2^ or 200 mg/m^2^) (TreoMel) between August 2019 and November 2023 at the University Hospital of Bern. We assessed treosulfan pharmacokinetics and correlations with several physiological parameters with potential impact on its interpatient variability. We further analyzed how treosulfan exposure correlates with toxicity and clinical outcomes. Women above 55 years showed higher area under the curve (AUC) levels (median: 946 mg*h/L, range: 776–1370 mg*h/L), as compared to women under 55 (median: 758 mg*h/L, range: 459–1214 mg*h/L, *p* = 0.0487). Additionally, women above 55 showed higher peak levels (median: 387 mg/L, range: 308–468 mg/L), as compared to men of the same age range (median: 326 mg/L, range: 264–395 mg/L, *p* = 0.0159). Treosulfan levels varied significantly with body temperature, liver enzymes, hemoglobin/hematocrit., and treosulfan exposure correlated with diarrhea severity in women over 55 (*p* = 0.0076). Our study revealed age- and gender-related variability in treosulfan pharmacokinetics, with higher plasma levels observed in female patients above 55. Moreover, our data suggest that treosulfan plasma levels may vary with several physiological parameters and that higher treosulfan exposure may impact toxicity. Our study underlines the need for further research on treosulfan pharmacokinetics, especially in older patients undergoing HDCT in the ASCT setting.

## 1. Introduction

Acute myeloid leukemia (AML) is a heterogeneous hematologic malignancy arising from clonally proliferating myeloid progenitor or stem cells, which expand in bone marrow, peripheral blood, and extramedullary tissues. AML is the most prevalent type of acute leukemia in adult patients, is diagnosed at a median age of 69, and is more common in men [1,2,3]. 

Curative approaches in fit patients with AML generally involve intensive induction therapy required to achieve maximal hematological remission, followed by consolidation therapy to prevent relapse. Patient characteristics including age, performance status, and comorbidities, along with the cytogenetic and molecular risk, determine disease prognosis and dictate the selection of optimal consolidation strategy [4,5,6,7,8]. High-dose chemotherapy (HDCT) followed by autologous stem cell transplantation (ASCT) can be implemented as a consolidation strategy in patients with favorable risk, as well as selected patients with minimal residual disease (MRD) negative intermediate-risk disease [9,10,11,12,13]. 

Recently, a number of studies have shown that treosulfan is an effective and safe myeloablative agent when used within pre-transplant conditioning regimens. Compared to busulfan-based regimens, treosulfan is associated with lower rates of liver, lung, and neurotoxicity, as well as less frequent irreversible alopecia [14,15,16,17,18,19].

However, despite dose adjustment by body surface area (BSA), substantial interpatient pharmacokinetic variability has been reported. In particular, the observed variability concerns the distribution at steady state (Vss), the total clearance (Cltot), and the area under the curve (AUC) [19,20,21,22,23,24,25,26,27,28]. As treosulfan is more commonly integrated into pre-transplant conditioning regimens, taking into account its pharmacokinetic features and variability becomes more crucial.

Treosulfan is a prodrug which undergoes a pH- and temperature-dependent, nonenzymatic sequential conversion into its monoepoxide and diepoxide with bifunctional alkylating activity, (2S,3S)-1,2-epoxy-3,4-butanediol 4-methanesulfonate (S,S-EBDM) and (2S,3S)-1,2:3,4-diepoxybutane (S,S-DEB), respectively [29,30,31]. Treosulfan is primarily eliminated through dose-dependent glomerular filtration, with an approximate 60% tubular reabsorption rate, along with additional pathways [19,24]. The half-life of treosulfan comprises 1.8 h [26,27,32].

Numerous efforts have been made to understand the determinants influencing Vss, Cltot, and AUC of treosulfan using population-based pharmacokinetic models. The distribution of treosulfan follows an open two-compartmental model characterized by first-order distribution and elimination kinetics [20,21,22,28,33]. So far, several physiological variables with potential impact on treosulfan pharmacokinetics have been tested, including age, sex, height, weight, BSA, renal function, use of diuretics, liver size, liver fibrosis, liver enzymes, serum ferritin, and hemoglobin levels [19,20,22,23,27,28,33]. Van den Berg et al. analyzed pharmacokinetic data from 93 adults and 23 children, finding only BSA as a covariate for the central and peripheral compartment volumes (V1 and V2) and Cltot. The BSA allometric scaling factor for Cltot was 1.29 (Cltot ∼ BSA1.29) [19,28].

The highest interpatient variability in Vss and Cltot was reported in children (when compared to adults). This was reported by the multicenter study by van der Stoep et al., where the coefficient of variation in pediatric patients reached up to 86% [19,20]. Although other studies have not found sex to be a significant covariate of treosulfan pharmacokinetics [20,22,23], our group previously reported higher peak and AUC values in women as compared to men [34]. On the contrary, rat models did show significant gender-related effects with faster elimination from plasma and organs in females as well as lower volume of distribution and higher systemic clearance compared to males [35,36]. Moreover, studies utilizing mathematical models or in vivo tests on rats, along with reviews exploring further factors including body temperature, blood pH, total body water (TBW), red blood cell (RBC) volume, and plasma levels, suggest potential effects of these variables on the distribution and clearance of treosulfan. However, these factors have not yet been investigated in humans [19,31,37,38].

The relevance of interindividual variability in treosulfan levels among adults, along with its underlying causes, remains understudied, particularly within pre-ASCT HDCT regimens in AML. In particular, it is unclear to what extent treosulfan levels, especially AUC, are related to outcome and toxicities [14,19,20]. The multicenter pediatric study of van der Stoep et al. [39] observed a correlation between higher treosulfan exposure (AUC > 1750 mg*h/L) and the incidence of skin toxicity and mucositis. In a previous study, we reported an association between AUC and peak values with duration of hospitalization [34].

As treosulfan is increasingly used in pre-transplant conditioning regimens, understanding its pharmacokinetics becomes crucial for improving the safety and outcomes of HDCT and ASCT. Hence, the aim of the present study was to investigate treosulfan pharmacokinetics and its interpatient variability in a cohort of adult patients with AML undergoing HDCT in the ASCT setting.

## 2. Results

### 2.1. Patient Characteristics

In this study, 55 patients were included, comprising 22 (40%) women and 33 (60%) men. Patient characteristics at diagnosis are summarized in Table 1. The cohort was divided into four subgroups according to the age at HDCT including women above 55 years (*n* = 8, median age: 66 years, range: 55–74 years), men above 55 years (*n* = 16, median age: 66 years, range: 57–73 years), women under 55 years (*n* = 14, median age: 47 years, range: 17–54 years), and men under 55 (*n* = 17, median age: 49 years, range: 23–54 years). The median age at HDCT of the entire patient cohort was 54 years (range: 17–74 years). Secondary AML was reported in one patient each in the group of men above 55 years and women under 55 years. Both patient cases were related to prior exposure to therapeutic drugs and/or radiation. 

### 2.2. Treatment Details

Treatment details are summarized in Table 2. All patients underwent HDCT followed by ASCT. 51 (93%) patients had received intensive induction therapy, while 5 (9%) patients had undergone alternative therapy, defined as any treatment not consisting of cytarabine monotherapy or cytarabine combination therapy such as the combination therapy with venetoclax and azacitidine or monotherapy with decitabine. After the second induction cycle, 45 (82%) patients had reached either complete remission (CR) or complete remission with incomplete hematologic recovery (CRi), and 13 (24%) achieved minimal residual disease (MRD)-negativity. All patients in the alternative therapy group reached CR or CRi, and 4 out of 5 patients achieved MRD-negativity. TreoMel 200 was administered to 22 (40%) patients, while 33 (60%) patients received TreoMel 140. After HDCT, 52 (95%) patients reached CR or CRi, and 22 (39%) achieved MRD-negativity. Following maintenance regimens have been administered depending on AML molecular profile: FLT3 Inhibitors (midostaurin 50 mg twice daily or gilteritinib 120 mg/day), administered for 12 months; IDH1/2 inhibitors (ivosidenib 500 mg/day or enasidenib 100 mg/day), for 2 years; or hypomethylating agents (azacitidine 75 mg/m^2^, d1 to 5, every 28 days; or decitabine 20 mg/m^2^, days 1 to 5, every 28 days) +/− venetoclax 100–400 mg/day, days 1 to 7–21 days every 28 days, administered for 6 cycles.

### 2.3. Treosulfan Plasma Concentration

**Figure 1:**
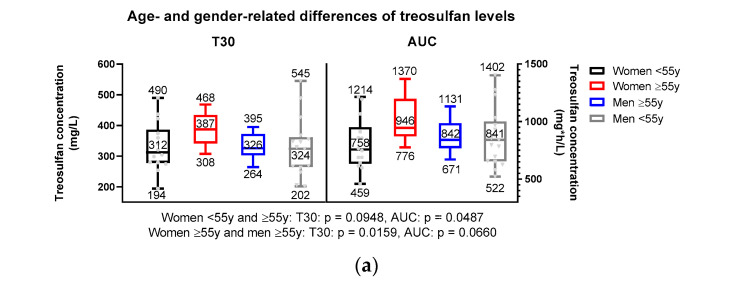

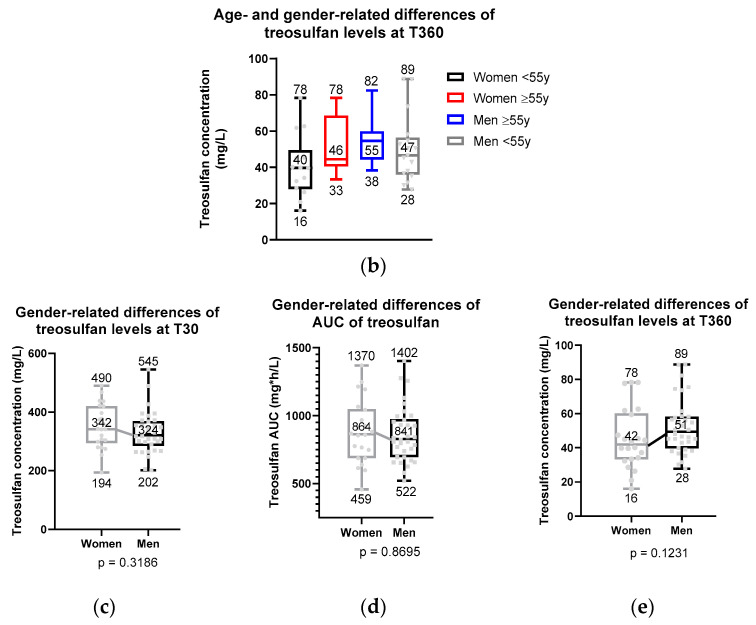
(**a**) Age- and gender-related differences of treosulfan levels at T30 and AUC. (**b**) Age- and related differences of treosulfan levels at T360. (**c**) Gender-related differences of treosulfan levels at T30 (**d**) AUC (**e**) and at T360.

Age and gender-related differences in peak level (T30), trough level (T360), and AUC of treosulfan are summarized in Table 3 and graphically illustrated in Figure 1. All treosulfan levels (T30, T60, T120, T240, T260, and AUC) regardless of age and gender are illustrated in Supplementary Figure S2.

The median AUC of the entire patient cohort was 855 mg*h/L (range: 459–1402 mg*h/L), while the mean AUC was 875 ± 213 mg*h/L. The median peak and trough levels were 334 mg/L (range: 194–545 mg/L) and 45 mg/L (16–89 mg/L), respectively. No significant differences in median peak levels and AUC were detected when comparing the entire cohort of women with all men, without considering age. Median peak levels and AUC were 342 mg/l (range: 194–490 mg/L) for women versus (vs.) 324 mg/L (range: 202–545 mg/L) for men, *p* = 0.3186 (Figure 1c), and 864 mg*h/L (range: 459–1370 mg*h/L) vs. 841 mg*h/L (range: 522–1402 mg*h/L), *p* = 0.8695 (Figure 1d), respectively. Median trough level was numerically lower in women, with 42 mg/L (range: 16–78 mg/L) vs. 51 mg/L (range: 28–89 mg/L) in men (*p* = 0.1231, Figure 1e).

A significant difference regarding peak level and AUC was observed for men above 55 years and a nearly significant difference in terms of AUC and peak level was identified for women above 55 years, with higher treosulfan levels in the female group. The median peak level in women above 55 years was 387 mg/L (range: 308–468 mg/L) vs. 326 mg/L (range: 264–395 mg/L) in men above 55 years (*p* = 0.0159, Figure 1a). Similarly, the median AUC in women above 55 years was 946 mg*h/L (range: 776–1370 mg*h/L) versus 842 mg*/L (range: 671–1131 mg*h/L) in men above 55 years (*p* = 0.0660, Figure 1a). 

Moreover, a significant difference in AUC was observed between women above 55 years and under the age of 55, with higher treosulfan levels in the older group: 946 mg*h/L (range: 776–1370 mg*h/L) vs. 758 mg*h/L (range: 459–1214 mg*h/L), *p* = 0.0487 (Figure 1a). Peak levels showed no significant differences among both groups (*p* = 0.0948).

A significant difference in trough levels was observed in women under 55 years with a median of 40 mg/L (range: 16–78 mg/L), compared to 55 mg/L (range: 38–82) in men above 55 years (*p* = 0.0187). No significant differences were found between the trough levels among the other subgroups (Figure 1b).

### 2.4. Correlations between Covariates and Treosulfan Levels

**Figure 2:**
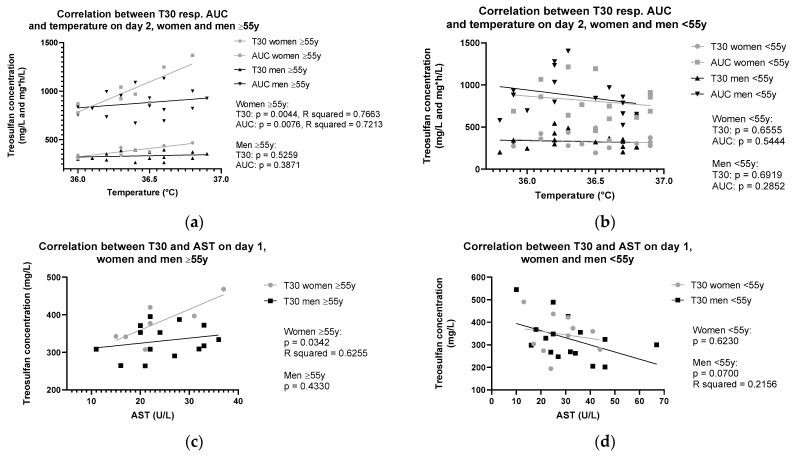

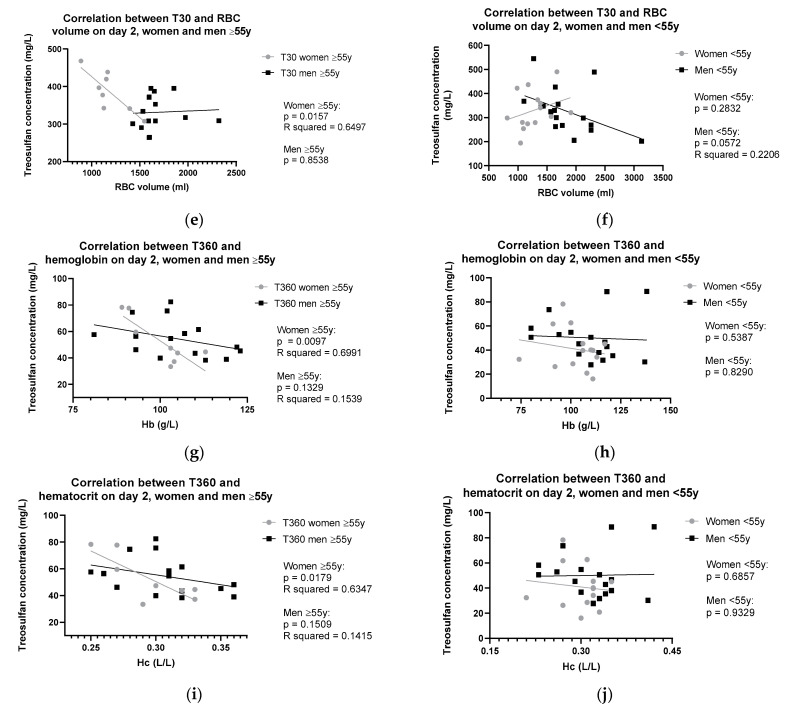
Correlation between treosulfan levels at T30 and T360, respectively, as well as AUC and vital signs or blood parameters. (**a**) Correlation between treosulfan levels at T30, T360 or AUC, respectively, and vital signs or blood parameters (**b**) and women and men under 55 y. (**c**) Correlation between T30 and aspartate aminotransferase (AST) measured on the first day of treosulfan application in women and men above 55 y (**d**) and women and men under 55 y. (**e**) Correlation between T30 and red blood cell (RBC) volume measured on the second day of treosulfan application in women and men above 55 y (**f**) and women and men under 55 y. (**g**) Correlation between T360 and hemoglobin (Hb) measured on the second day of treosulfan application in women and men above 55 y (**h**) and women and men under 55 y. (**i**) Correlation between T360 and hematocrit (Hc) measured on the second day of treosulfan application in women and men above 55 y (**j**) and women and men under 55 y.

Significant correlations with R-squared values above 0.6 between treosulfan levels and physiological parameters are summarized in Figure 2. Of note, parameter values were obtained on both the first and second day of treosulfan administration, and whenever possible, the day with the most reported values was chosen to correlate the values with treosulfan peak level, trough level, and AUC. 

In women above 55 years, a significant positive correlation was observed, respectively, between peak levels and AUC and body temperature (T30: *p* = 0.0044, R-squared value = 0.7663; AUC: *p* = 0.0076, R-squared value = 0.7213, Figure 2a). Additionally, within the same subgroup, peak levels correlated with AST levels (*p* = 0.0342, R-squared value = 0.6255, Figure 2c) and RBC volume (*p* = 0.0157, R-squared value = 0.6497, Figure 2e). Furthermore, in women above 55 years, negative correlations between trough levels and hemoglobin (*p* = 0.0097, R-squared value = 0.6991, Figure 2g), as well as hematocrit (*p* = 0.0179, R-squared value = 0.6347, Figure 2i) were observed.

In men under 55 years, nearly significant negative correlations were found between peak levels and AST (*p* = 0.0700, R-squared value = 0.2156, Figure 2d) as well as RBC volume (*p* = 0.0572, R-squared value = 0.2206, Figure 2f). Similarly, in men above 55 years, nearly significant negative correlations with low R-squared values were observed between trough levels and hemoglobin (*p* = 0.1329, R-squared value = 0.1539) and hematocrit (*p* = 0.1509, R-squared value = 0.1415). When adjusting RBC by body weight or BSA, significant correlations with T30, T360, and AUC were only found for women over the age of 55. 

No significant correlations between weight, height, body mass index (BMI), BSA, TBW volume and percentage, plasma volume and percentage, RBC percentage, total blood volume, glomerular filtration rate (GFR), potassium, alanine transaminase (ALT) levels, and treosulfan levels were found.

The median and ranges of all significant parameters are listed in descending order in the following paragraph. The median temperature was 36.5 °C (36–36.9 °C) in men above 55 years, 36.5 °C (35.9–36.9 °C) in women under 55 years, 36.4 °C (36–36.8 °C) in women above 55 years, and 36.3 °C (35.8–36.8 °C) in men under 55 years. The median AST was 29 U/L (10–67 U/L) in men under 55 years, 28 U/L (13–44 U/L) in women under 55 years, 24 U/L (11–36 U/L) in men above 55 years, and 22 U/L (15–37 U/L) in women above 55 years. The median RBC volume was 1662 mL (1104–3131 mL) in men under 55 years, 1540 mL (1296–2239 mL) in men above 55 years, 1237 mL (816–1912 mL) in women under 55 years, and 1139 mL (889–1547 mL) in women above 55 years. The median hemoglobin level was 110 g/L (138–80 g/L) in men under 55 years, 106 g/L (74–117 g/L) in women under 55 years, 103 g/L (81–123 g/L) in men above 55 years, and 103 g/L (89–113 g/L) in women above 55 years. The median hematocrit level was 0.33 L/L (0.23–0.42 L/L) in men under 55 years, 0.32 L/L (0.21–0.35 L/L) in women under 55 years, 0.31 L/L (0.25–0.36 L/L) in men above 55 years, and 0.30 L/L (0.25–0.33 L/L) in women above 55 years.

### 2.5. Adverse Events

The most frequently observed toxicities after HDCT are summarized in Table 4. Details concerning malnutrition, the requirement for parenteral nutrition, and infections during hospitalization are provided in Supplementary Tables S1 and S2, respectively.

In all subgroups, the toxicities demonstrated a balanced distribution, with no significant differences observed among the different groups. The most frequently reported adverse effects were pancytopenia, observed in all patients. Diarrhea of grade I to III severity occurred in 54 (98%) patients, while nausea was reported in 45 (82%) individuals. Oral mucositis occurred in 32 (58%) patients, with severity ranging from grade I to IV. Engraftment syndrome was reported in 10 (18%) patients. Hepatopathy was documented in 2 (4%) cases, presenting either as self-limiting drug-induced liver injury (DILI) or acute hepatopathy. No epileptic seizures were reported.

**Figure 3:**
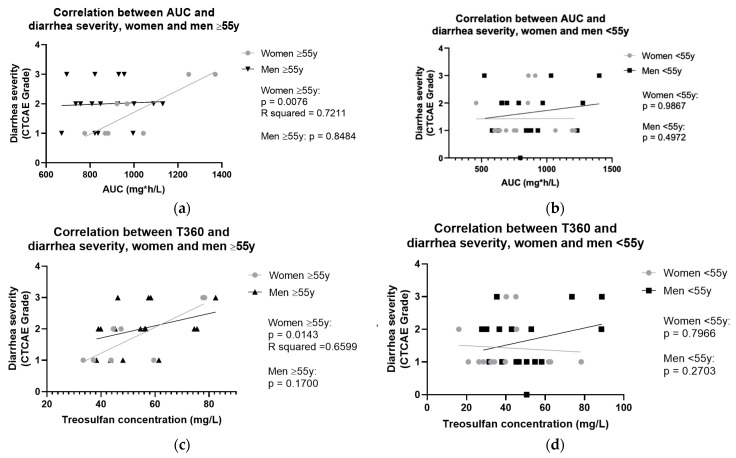
Correlation between treosulfan levels at T360 or AUC and diarrhea severity after high-dose chemotherapy. (**a**) Correlation between AUC and diarrhea severity in women and men above 55 y (**b**) and women and men under 55 y. (**c**) Correlation between T360 and diarrhea severity in women and men above 55 y (**d**) and women and men under 55 y. Abbreviations: CTCAE, Common Terminology Criteria for Adverse Events.

Significant correlations with R-squared values above 0.6 between treosulfan levels and toxicities are summarized and compared among all subgroups in Figure 3. 

A significant correlation between treosulfan levels and toxicity was found in women above 55 years. In this subgroup, AUC and trough levels correlated with diarrhea severity (AUC: *p* = 0.0076, R-squared values = 0.7211, T360: *p* = 0.0143, R-squared value = 0.6599). In the remaining subgroups, no significant causally explicable correlations were identified.

### 2.6. Hematologic Response and Clinical Outcome

Hematologic engraftment and clinical outcome are summarized in Table 5. Survival analyses are provided in the Supplementary Figure S1.

The median follow-up duration for all patients from ASCT was 17 months (range: 0.5–46 months). The median time from AML diagnosis to ASCT was 3 months (range: 2–7 months). Regarding hematologic response, no significant differences in engraftment duration were observed among the different subgroups.

The median duration of hospitalization was 22 days (range: 15–101 days), with numerically the longest duration in women above 55 years (26 days, range: 21–34 days, *p* = 0.9020). A total of 22 (40%) relapses were documented, with a median time to relapse of 5 months (range: 1–36 months). Additionally, 18 (33%) deaths occurred, with a median time to death of 7 months (range: 0.5–49 months). In all subgroups, no significant correlation between treosulfan levels and hematologic response, hospitalization duration, or relapse occurrence was identified. The cumulative incidence of post-transplant relapse was 0.343 (95% CI: 0.204–0.502) at 6 months, 0.469 (95% CI: 0.334–0.637) at 12 months, and 0.498 (95% CI: 0.359–0.657) at 24 months.

Concerning PFS and OS, the log-rank (Mantel-Cox) test showed no differences among all patient subgroups (PFS: *p* = 0.1645 and OS: *p* = 0.4340). The median PFS time of women above 55 years was 5 months (95% CI: 3–not reached), compared to 36 months (95% CI: 5–not reached) in men above 55 years, 10 months (95% CI: 7–ot reached) in men under 55 years, and undefined in women under 55 years. The median OS of women above 55 years was 7 months (95% CI: 6–not reached), compared to 49 months (95% CI: 13–not reached) in men above 55 years. The median OS time of women and men under 55 years was undefined. The largest difference in PFS was observed between women above 55 years and men under 55 years (PFS: *p* = 0.0430), while there was no statistically significant difference regarding OS (*p* = 0.1247).

## 3. Discussion

Over the last decade, numerous studies have shown that treosulfan is an active and well-tolerated myeloablative drug when used in pre-transplant conditioning regimens. In contrast to the more frequently used busulfan-based HDCT regimens, treosulfan offers the benefit of a milder toxicity profile, particularly lower rates of early liver, lung, and neurotoxicity, as well as low rates of irreversible alopecia [14,15,16,17,18,19]. Considerable interpatient variability in treosulfan pharmacokinetics has been reported in previous studies, yet the underlying causes for this heterogeneity are not fully elucidated. Moreover, previous research in this context has primarily focused on pediatric cohorts [19]. To the best of our knowledge, this study represents the largest adult cohort examining interindividual pharmacokinetic variability of treosulfan levels, in the context of HDCT in fit AML patients. Our data suggest that treosulfan pharmacokinetics are gender- and age-dependent, with the highest peak and AUC values observed in women above the age of 55. Moreover, our study explored the impact of further physiological parameters with potential effects on treosulfan interpatient variability. We additionally analyzed how treosulfan pharmacokinetics correlate with safety parameters and clinical outcomes. Our results suggest that higher treosulfan exposure might correlate with increased toxicity, specifically the severity of diarrhea. 

In this study, the mean AUC (±standard deviation) among patients receiving 14 g/m^2^/d treosulfan was 875 ± 213 mg*h/L. Our AUC values were numerically lower than the AUC reported in the literature: Beelen et al. [25] reported 1104 ± 173 mg*h/L (median patient age: 51 years), Nemecek et al. [26] documented 1309 ± 262 mg*h/L (median age: 34 years), Mohanan et al. [23] reported 1396 ± 715 mg*h/L (median age: 9 years), and Glówka et al. [27] documented 2400 ± 1267 mg*h/L (median age: 7.5 years). Discrepancies among these studies may result from heterogeneity in cohort size, as well as gender and age distribution [19,34].

Exclusively focusing on gender, we observed numerically higher median peak levels and AUC values in women, along with numerically higher median trough levels in men. Considering age, we observed higher AUC values and peak levels in women above the age of 55. Previous data suggest that age-related variabilities may stem from pharmacokinetic changes over the course of life, particularly related to TBW and modifications in renal function [19]. As a consequence of decreasing TBW with older age, water-soluble agents like treosulfan are expected to have a lower volume of distribution, resulting in higher plasma levels among older individuals [19,40,41,42]. Furthermore, age-related decrease in GFR, considered a physiological process after 30–40 years of age, intensifies notably after 70, potentially leading to drug accumulation [43,44,45]. Comparable gender-related differences may result in variations in treosulfan levels, characterized by generally lower TBW, GFR, and tubular reabsorption rates in women [42,46]. In our study, no correlations between TBW and treosulfan levels were found. The higher trough levels in men compared to women might be explained by findings from rat models, which indicate that gender significantly impacts the elimination constant and systemic clearance of treosulfan. Females have a faster elimination from plasma and organs, along with higher systemic clearance compared to males [35,36]. Given that our cohort primarily comprised patients undergoing intentionally curative treatment, GFR restrictions were minimal, warranting investigations in further studies. 

Other physiologic variables explored in the literature include height, weight, BSA, co-medication with diuretics, liver size, liver fibrosis, liver function, as well as ferritin and hemoglobin levels [19,20,22,23,27,28,33]. Among these, only BSA was found to treosulfan volumes of distribution and clearance, with higher BSA leading to increased Cltot [28]. In our work, significant correlations with body temperature, AST, RBC, and hemoglobin/hematocrit were exclusively found in the subgroup of women above the age of 55.

Pharmacokinetic changes related to body temperature may be attributed to the process in which the prodrug treosulfan undergoes a pH- and temperature-dependent, nonenzymatic sequential conversion into its active epoxides [29,30,31]. Romański et al. observed that modification in body temperature of 1 °C results in a 17% increase in the epoxide conversion rate constant, suggesting that body temperature could be a significant determinant of Cltot and epoxide clearance [37]. Kinetic studies indicate that treosulfan elimination primarily proceeds via epoxide conversion [31]. Consequently, higher body temperature is expected to enhance epoxide conversion and elimination, resulting in decreased treosulfan levels. Within our dataset, we observed that treosulfan concentrations rose with increasing body temperature in women above 55 years. However, in our study, median body temperature did not vary significantly within our patient cohort, possibly due to the single morning measurement. Therefore, future studies should ideally assess body temperature multiple times a day providing an average value. 

Previous data from both in vitro and in vivo animal studies on epoxides suggest that liver and lung metabolism via epoxide hydrolases are the primary routes to eliminating the epoxy metabolites of treosulfan [47,48,49]. The negligible first-pass effect indicates that the parent drug treosulfan undergoes no hepatic metabolism [19,47]. In our study, we found a significant correlation between AST and peak values in women above 55 years, indicating higher treosulfan levels with rising AST levels. Conversely, a negative correlation was observed in men under 55 years. No correlation with ALT levels was observed. Considering the median AST values in all subgroups, women under 55 years have the lowest AST values, while men under 55 years have the highest. Further studies in larger patient cohorts would be needed to assess the correlation with liver enzyme levels.

Furthermore, we report negative correlations between peak values and RBC volume, as well as between trough levels and hematocrit or hemoglobin in women above 55 years. Considering all subgroups, in women above the age of 55, the lowest median hematocrit (0.30 L/L) and hemoglobin (103 g/L) values were observed, showing a statistically significant correlation with the T360 value (*p* = 0.0179 and *p* = 0.0097, respectively). In men under 55, the highest median hematocrit (0.33 L/L) and hemoglobin (110 g/L) values were observed; however, these values did not correlate with the T360 level (*p* = 0.9329 and *p* = 0.8290, respectively). This correlation is consistent with a previous study that observed higher concentrations of treosulfan and its epoxides in plasma compared to whole blood or RBC [38].

Regarding safety profile, we observed a correlation between AUC and trough levels with severity of diarrhea. Higher treosulfan values associated with increased diarrhea severity in women above 55 years. This finding is consistent with the multicenter pediatric studies of van der Stoep et al. [20,39], observing that elevated treosulfan exposure (AUC > 1650 mg*h/L [20] and >1750 mg*h/L [39]) correlated with an increased risk of mucosal toxicity. 

Hematologic recovery and duration of hospitalization were similar among all patient subgroups. We observed significant differences in PFS between women above 55 years and men under 55 years, while there were no statistically significant differences in OS. Previous studies showed no correlation between treosulfan exposure and OS or PFS [20,39]. The main limitations of our study are the retrospective single-center study design, the relatively small sample size, gender imbalance, short follow-up time, and the potential impact of multiple confounders. Therefore, the distinct correlations reported are uniquely hypothesis-generating, and larger studies would be required to confirm these findings. 

## 4. Materials and Methods

### 4.1. Study Design, Patient Cohort and Endpoints

This single-center retrospective study analyzed a cohort of adult AML patients considered fit to receive HDCT with treosulfan and melphalan (TreoMel) prior to ASCT. Patients were considered fit for HDCT if they were =/<75 years old, had an ECOG performance status of 0–1, had no major comorbidities, and an adequate organ function. Patients underwent HDCT between August 2019 and November 2023 at the University Hospital of Bern, Switzerland.

The main endpoint of the study was to assess age and gender-related variability of treosulfan plasma levels, including peak and trough levels, as well as AUC. Secondary endpoints were to correlate treosulfan plasma levels with selected physiological parameters, as well as adverse events and clinical outcomes in terms of progression-free survival (PFS) and overall survival (OS).

The study was conducted following the guidelines of the Declaration of Helsinki, and approved by the local ethics committee of Bern, Switzerland (decision number 2024-00929).

### 4.2. Treatment and HDCT Schedule

Patients received between one and two cycles of induction chemotherapy. If patients achieved complete remission, induction was followed by stem cell mobilization and apheresis, with subsequent HDCT with TreoMel, and ASCT on day 0. Treosulfan was administered at a dose of 14 g/m^2^/d for three days, and melphalan at a dose of either 140 mg/m^2^ (TreoMel 140), administered on one day, or 200 mg/m^2^ (TreoMel 200), administered as split-dose over two days. With TreoMel 140, patients received treosulfan on days −4, −3, and −2 followed by melphalan on day −1. With TreoMel 200, treosulfan was given on days −5, −4, −3, and subsequently, 100 mg/m^2^ melphalan on days −2 and −1. Both agents were administered intravenously: treosulfan over two hours in a 5% glucose solution and melphalan over one hour in a 0.9% NaCl solution. 

As prophylactic anti-infective therapy, patients received valaciclovir, sulfamethoxazole-trimethoprim, and fluconazole. Prior to ASCT, anti-allergic prophylaxis with methylprednisolone and clemastine was administered. Furthermore, dexamethasone was given to prevent engraftment syndrome starting on day +8 [50], and allopurinol was administered during HDCT as hyperuricemia prophylaxis. As supportive co-medication, patients received antiemetics, proton pump inhibitors, anticoagulants, and filgrastim. Folic acid was administered for 8 weeks after ASCT to support hematologic recovery.

### 4.3. Treosulfan Pharmacokinetics and Calculations

To assess treosulfan levels, blood samples were collected on the second day of the treosulfan schedule following a standardized protocol. The first blood sample was obtained before the start of treosulfan infusion (defined as T0), afterwards five follow-up samples were collected at 30, 60, 120, 240, and 360 min after the stop of treosulfan infusion (termed as T30, T60, T120, T240, T360). Blood sample levels obtained at 30 min were defined as peak levels, while those after 360 min were designated as trough levels. Treosulfan exposure (AUC_T0−T360_) was calculated with the measured drug concentrations using the trapezoid rule of GraphPad Prism^®^ 8.0.1 [51]. When measuring T0, the laboratory’s measurement threshold of <20 mg/L was reached, for this reason, a uniform lower value for T0 of 20 mg/L was selected for the calculation of AUC.

For drug-level analysis, ultra-high performance liquid chromatography-tandem mass spectrometry (UHPLC-MS/MS) was performed. Mass spectrometric measurements were obtained using multiple reaction monitoring on Xevo TQ-S (Waters Corp., Milford, MA, USA) [14,34]. Immediately during blood collection, samples were stabilized with a sodium citrate buffer and stored at a temperature of −80 °C until analysis. As previously described [14,34], individual solutions of treosulfan and the internal standard (^2^H_4_)-treosulfan (w = 95%; Alsachim; Illkirch Graffenstaden, France) were prepared, along with 6 calibrator-spiking solutions with concentrations of 2.8, 5.6, 11.3, 22.5, 45, and 90 mg/L. For UHPLC-MS/MS analysis, the samples were precipitated, centrifuged and the supernatant finally diluted corresponding to the calibration range. After that 0.5 μL of each prepared sample was injected into a reverse-phase CORECTS UPLC T3 column and chromatographically resolved over 3.0 min. Subsequently, the analytes were introduced by means of the eluent into the mass spectrometer (Xevo TQ-S, Waters Corp., Milford, MA, USA), operating in positive ion electrospray ionization mode (ESI+), as previously described [14,34]. Data processing and analysis were performed using TargetLynx (MassLynx software, version 4.1, Waters Corp., Milford, MA, USA), by integrating the area under the specific multiple reactions monitoring chromatograms in reference to the area of the isotope-labeled analog [14,34].

### 4.4. Covariates

The selection of covariates was based on findings from previous studies and on parameters suggested to potentially correlate with interpatient variability [19,20,22,23,27,28,31,33,37,38]. These parameters included age at HDCT, gender, weight, height, BMI, BSA, body temperature, TBW volume and percentage, hemoglobin, hematocrit, plasma volume and percentage, RBC volume and percentage, total blood volume, glomerular filtration rate (GFR), along with potassium, aspartate aminotransferase (AST), and alanine transaminase (ALT) levels. Measurements were performed on both the first and second day of treosulfan administration, and whenever possible, the day with the most reported values was chosen to separately correlate the values with peak level, trough level, and AUC. BSA was calculated using the Dubois formula [52]. For TBW, Watson’s formula was used [53]. RBC, plasma, and total blood volume were estimated with Nadler’s formula [54]. Weight and body temperature, measured in the morning, were collected from daily follow-up reports by nursing staff. Height was measured once at the initiation of the main treatment regimen.

### 4.5. Assessment of Adverse Events, Hematologic Recovery and Survival Rates

Information on adverse events was gathered by analyzing laboratory and microbiology reports as well as daily follow-up reports of doctors and nurses. Adverse events severity was evaluated using version 5.0 of the Common Terminology Criteria for Adverse Events (CTCAE) developed by the National Cancer Institute [55]. Additionally, hematologic recovery after ASCT was assessed based on the duration of cytopenia, particularly thrombocytopenia and neutropenia. 

Relapse rates, PFS, and OS were evaluated using data from reports collected during follow-up visits of patients in the outpatient department of the clinic. Relapse was defined by an increase in bone marrow blasts exceeding 5% (cytomorphologic relapse) or the detection of molecularly detectable disease (MRD-positivity) in bone marrow or peripheral blood. MRD was assessed for the first time 3–4 weeks post-ASCT. PFS was determined as survival time without evidence of progression, relapse, or death, whichever occurred first. The time from autograft to the last follow-up or death, regardless of cause, was defined as OS. 

### 4.6. Statistical Analysis

The data cut-off date was 1 January 2024. Statistical analysis and graphical representations were conducted using GraphPad Prism^®^ 8.0.1. Kaplan-Meier survival analysis was performed to calculate and graphically visualize PFS and OS. An unpaired *t*-test was utilized to compare treosulfan levels and AUC of the subgroups. The Pearson correlation coefficient was used to investigate the correlation between treosulfan levels, physiological parameters, toxicity, and hematologic outcome. Values and percentages were rounded to the nearest whole number. A *p*-value below 0.05 was used in order to reject the null hypothesis. The threshold for R-squared values in linear regression analysis was defined as greater than 0.6. The age cutoff for the subgroup analysis was selected based on the median age of the study population.

## 5. Conclusions

We observed considerable interpatient pharmacokinetic variability in treosulfan plasma levels in adult patients with AML undergoing HDCT previous to ASCT. Our work suggests that treosulfan plasma levels are gender- and age-dependent, with higher levels observed in female patients above the age of 55. Multiple physiological variables might contribute to interpatient variations in tresosulfan pharmacokinetics, such as differences in body temperature, liver enzymes, RBC volume, hematocrit, and hemoglobin levels. Further studies should explore the impact of these factors on a larger patient population. Furthermore, in our patient cohort higher treosulfan exposure correlated with the severity of diarrhea. Therefore, therapeutic drug monitoring, especially in older patients, should be considered in the HDCT settings. Larger multicentric studies should be conducted to confirm our results, aiming to optimize treatment regimens and improve outcomes of HDCT and ASCT in AML patients.

## Figures and Tables

**Table 1:** Patient characteristics at diagnosis.

Characteristics	Women ≥ 55 y (*n* = 8)	Men ≥ 55 y (*n* = 16)	Women < 55 y (*n* = 14)	Men < 55 y (*n* = 17)	All (*n* = 55)	*p*-Value
Median age at diagnosis, years (range)	65 (54–74)	65 (56–73)	47 (17–54)	49 (23–54)	54 (17–74)	<0.0001
ELN risk categories						
favorable	4 (50%)	6 (38%)	6 (43%)	9 (53%)	25 (46%)	0.8522
intermediate	2 (25%)	1 (6%)	6 (43%)	3 (18%)	12 (22%)	0.1075
adverse	2 (25%)	9 (53%)	2 (14%)	5 (29%)	18 (33%)	0.0997
FAB classification						
M0	1 (13%)	2 (12%)	1 (7%)	0	4 (7%)	0.4736
M1	1 (13%)	4 (24%)	6 (43%)	5 (29%)	16 (29%)	0.5171
M2	4 (50%)	3 (18%)	1 (7%)	6 (35%)	14 (26%)	0.0936
M3	0	0	0	0	0	1.0
M4	2 (25%)	6 (38%)	5 (36%)	5 (29%)	18 (33%)	0.9369
Secondary AML						
th-AML	0	1 (6%)	1 (7%)	0	2 (4%)	0.6566
MDS/MPD related	0	0	0	0	0	1.0
Peripheral Blood Parameters mean (range)						
Hemoglobin, g/dL	84 (±17)	99 (±20)	88 (±25)	82 (±30)	89 (±25)	0.2309
WBC, G/L	50 (±40)	24 (±38)	39 (±69)	67 (±80)	45 (±63)	0.2729
Platelets, G/L	58 (±42)	90 (±58)	108 (±76)	91 (±47)	90 (±59)	0.2869
Peripheral blasts, %	49 (±37)	33 (±30)	40 (±36)	48 (±32)	42 (±33)	0.5849
BM blasts, %	74 (±21)	73 (±25)	72 (±22)	78 (±22)	74 (±23)	0.8674
LDH, U/L	717 (±348)	672 (±527)	757 (±1230)	925 (±764)	778 (±800)	0.8318

Abbreviations: y, years; ELN, European LeukemiaNet genetic risk stratification; FAB, French American-British classification; AML, acute myeloid leukemia; MDS, Myelodysplastic syndrome; MPD, Myeloproliferative disorders; WBC, white blood cells; BM, bone marrow; LDH, lactate dehydrogenase; th-AML, related to prior exposure to therapeutic drugs and/or radiation.

**Table 2:** Induction treatment regimens and high-dose chemotherapy with autologous stem cell transplantation.

Characteristics	Women ≥ 55 y (*n* = 8)	Men ≥ 55 y (*n* = 16)	Women < 55 y (*n* = 14)	Men < 55 y (*n* = 17)	All (*n* = 55)	*p*-Value
Induction therapy	6 (75%)	14 (88%)	14 (100%)	17 (100%)	51 (93%)	0.0490
Cycles, (*n*)						
1	0	0	0	1 ^G^ (6%)	1 (2%)	1.0
2	6 (75%)	13 (81%)	14 (100%)	16 (94%)	49 (89%)	0.1743
3	0	1 ^F^ (6%)	0	0	1 (2%)	0.6909
Remission status after the first induction cycle						
CR	2 (25%)	3 (19%)	7 (50%)	5 (29%)	17 (31%)	0.3292
CRi	3 (38%)	7 (44%)	5 (36%)	10 (59%)	25 (46%)	0.5751
Other ^E^	1 (13%)	4 (25%)	2 (14%)	2 (12%)	9 (16%)	0.7882
MRD status after the first induction cycle						
MRD-positive ^J^	6 (75%)	12 (75%)	12 (86%)	14 (82%)	44 (80%)	0.8766
MRD-negative ^J^	0	2 (13%)	2 (14%)	3 (18%)	7 (13%)	0.8153
Remission status after second induction cycle ^A^						
CR	1 (13%)	2 (13%)	3 (21%)	4 (24%)	10 (18%)	0.8365
CRi	3 (38%)	11 (69%)	9 (64%)	12 (71%)	35 (64%)	0.4480
Other ^E^	2 (25%)	1 (6%)	2 (14%)	0	5 (9%)	0.1048
MRD status after second induction cycle ^A^						
MRD-positive ^J^	5 (63%)	12 (75%)	9 (64%)	11 (65%)	37 (67%)	0.8791
MRD-negative ^J^	1 (13%)	2 (13%)	5 (36%)	5 (29%)	13 (24%)	0.4249
Alternative therapy ^B^	2 (25%)	2 (13%)	0	1 ^G^ (6%)	5 (9%)	0.2316
Best remission response ^C^						
CR	2 (25%)	1 (6%)	0	0	3 (6%)	0.0480
CRi	0	1 (6%)	0	1 (6%)	2 (4%)	1.0
Best MRD response ^C^						
MRD-positive ^J^	0	0	0	1 (6%)	1 (2%)	1.0
MRD-negative ^J^	2 (25%)	2 (13%)	0	0	4 (7%)	0.0490
HDCT: TreoMel 200 ^H^	4 (50%)	5 (31%)	8 (57%)	5 (29%)	22 (40%)	0.3427
HDCT: TreoMel 140 ^I^	4 (50%)	11 (69%)	6 (43%)	12 (71%)	33 (60%)	0.3427
Remission status after HDCT						
CR	0	0	1 (7%)	3 (18%)	15 (27%)	0.2636
CRi	7 (88%)	14 (88%)	13 (93%)	14 (82%)	37 (67%)	0.9415
Other ^E^	1 (13%)	0	0	0	1 (2%)	0.0408
No BM puncture ^D^	0	2 (13%)	0	0	2 (4%)	0.2364
MRD status after HDCT						
MRD-positive ^J^	6 (75%)	9 (56%)	6 (43%)	10 (59%)	31 (56%)	0.5672
MRD-negative ^J^	2 (25%)	5 (31%)	8 (57%)	7 (41%)	22 (39%)	0.4248
No BM puncture ^D^	0	2 (13%)	0	0	2 (4%)	0.2364

Abbreviations: CR, complete remission; CRi, complete remission with incomplete hematologic recovery; MRD, minimal residual disease; BM, bone marrow; HDCT, High-dose chemotherapy. ^A^: In the case of induction therapy with two or more cycles. ^B^: Any therapy not consisting of cytarabine monotherapy or cytarabine combination therapy. ^C^: In the case of alternative therapy. ^D^: No bone marrow puncture due to critical general condition followed by death. ^E^: Not defined as CRi or CR: Any remission status with >5% of blasts either in bone marrow histology or if not representative in bone marrow aspirate. ^F^: Third induction cycle due to participation in a clinical trial. ^G^: No second induction cycle was administered due to multiple complications after first induction cycle, after first induction cycle an alternative therapy was given for the next cycles. ^H^: Cumulative dose of melphalan 200 mg/m^2^, treosulfan 42 g/m^2^. ^I^: Cumulative dose of melphalan 140 mg/m^2^, treosulfan 42 g/m^2^. ^J^: MRD assessment by flow cytometry and molecular genetics.

**Table 3:** Details of treosulfan plasma concentration.

Median ^A^, mg/L, mg*h/L (Range)	Women ≥ 55 y (*n* = 8)	Men ≥ 55 y (*n* = 16)	Women < 55 y (*n* = 14)	Men < 55 y (*n* = 17)	All Patients (*n* = 55)	All Women (*n* = 22)	All Men (*n* = 33)	*p*-Value
T30 ^B^	387 (308–468)	326 (264–395)	312 (194–490)	324 (202–545)	334 (194–545)	342 (194–490)	324 (202–545)	0.2652
T360 ^C^	46 (33–78)	55 (38–82)	40 (16–78)	47 (28–89)	45 (16–89)	42 (16–78)	51 (28–89)	0.1387
AUC	946 (776–1370)	842 (671–1131)	758 (459–1214)	841 (522–1402)	855 (459–1402)	864 (459–1370)	841 (522–1402)	0.2010

Abbreviations: AUC, the area under the curve. ^A^: Median treosulfan plasma concentration. ^B^: T30: Treosulfan plasma concentration measured 30 min after treosulfan application on the second day. ^C^: T360: Treosulfan plasma concentration measured 360 min after treosulfan application on the second day.

**Table 4:** Toxicities.

Characteristics Toxicity, *n* (%)	Women ≥ 55 y (*n* = 8)	Men ≥ 55 y (*n* = 16)	Women < 55 y (*n* = 14)	Men < 55 y (*n* = 17)	All (*n* = 55)	*p*-Value
Diarrhea	8 (100)	16 (100)	14 (100)	16 (94)	54 (98)	1.0
Grade I	4 (50)	4 (25)	10 (71)	7 (41)	25 (46)	0.0837
Grade II	2 (25)	8 (50)	2 (14)	6 (35)	18 (33)	0.2212
Grade III	2 (25)	4 (25)	2 (14)	3 (18)	11 (20)	0.8766
Grade IV	0	0	0	0	0	1.0
Oral Mucositis	4 (50)	10 (63)	11 (79)	7 (41)	32 (58)	0.1963
Grade I	2 (25)	4 (25)	3 (21)	4 (24)	13 (24)	1.0
Grade II	0	2 (13)	3 (21)	3 (18)	8 (15)	0.6659
Grade III	2 (25)	3 (19)	5 (36)	0	10 (18)	0.0373
Grade IV	0	1 (6)	0	0	1 (2)	0.6909
Nausea	7 (88)	12 (75)	12 (86)	14 (82)	45 (82)	0.8457
Headache	0	2 (13)	4 (29)	2 (12)	8 (15)	0.3703
Fatigue	6 (75)	7 (13)	7 (50)	6 (25)	26 (47)	0.3424
Pain	6 (75)	10 (63)	14 (100)	16 (94)	46 (84)	0.0141
Pancytopenia	8 (100)	16 (100)	14 (100)	17 (100)	55 (100)	-
Electrolyte imbalance	4 (50)	9 (56)	8 (57)	10 (59)	31 (56)	1.0
Engraftment syndrome	2 (25)	1 (6)	3 (21)	4 (24)	10 (18)	0.4944
Paroxysmal tachycardic episode	2 (25)	2 (13)	0	0	4 (7)	0.0490
Hepatopathy ^A^	0	1 (6)	0	1 (6)	2 (4)	1.0
Epileptic seizure	0	0	0	0	0	1.0

^A^: Self-limiting Drug-induced liver injury (DILI) or acute hepatopathy.

**Table 5:** Hematologic engraftment and clinical outcome.

Median Time ^A^ (Range)	Women ≥ 55 y (*n* = 8)	Men ≥ 55 y (*n* = 16)	Women < 55 y (*n* = 14)	Men < 55 y (*n* = 17)	All (*n* = 55)	*p*-Value
Follow up, m	7 (3–39)	20 (0.5–44)	8 (0.5–46)	21 (1–42)	17 (0.5–46)	0.4255
TTI ^B^, m	3 (2–6)	4 (3–7)	3 (2–4)	3 (2–7)	3 (2–7)	0.1220
CD34+, *n* × 10⁶/kg ^C^	3.613 (2.055–8.138)	4.094 (2.816–10.84)	3.422 (2.072–6.670)	3.446 (2.178–10.46)	3.621 (2.055–10.84)	0.3554
ANC ≥ 0.5 G/L, d	11 (10–13)	11 (8–13)	12 (11–13)	12 (10–14)	11 (8–14)	0.0072
ANC ≥ 1.0 G/L, d	11.5 (11–13)	12 (10–112)	13 (11–17)	12 (10–14)	12 (10–112)	0.5921
PLT > 20 G/L, d	29 (13–82)	19 (14–182)	21.5 (13–56)	27 (14–92)	23 (13–182)	0.6648
PLT > 100 G/L, d	62.5 (32–186)	75.5 (36–351)	66 (18–566)	70 (33–413)	75 (18–566)	0.8688
Hospitalization, d	26 (21–34)	21 (18–60)	21.5 (15–40)	21 (19–101)	22 (15–101)	0.9020
Relapse, *n* (%)	5 (63)	6 (38)	4 (29)	7 (41)	22 (40)	0.5177
Relapse, m	3 (2–5)	5.5 (2–12)	5 (1–19)	5 (3–10)	5 (1–19)	0.5080
Deaths, *n* (%)	4 (50)	7 (44)	2 (14)	5 (29)	18 (33)	0.2243
Death, m	6.5 (5–7)	12 (0.5–49)	6.5 (4–9)	14 (7–28)	7 (0.5–49)	0.5890

Abbreviations: m, months; d, days; TTI, Time to treatment initiation; ANC, absolute neutrophil count; PLT, platelets. ^A^: Time since autologous stem cell transplantation. ^B^: Defined as time from diagnosis to initiation of autologous stem cell transplantation. ^C^: At autologous stem cell transplantation.

## Data Availability

The data presented in this study are available upon request from the corresponding author.

## Supplementary Materials

The following supporting information can be downloaded at: https://www.mdpi.com/article/10.3390/ijms25158215/s1.
