# Supplementary material for: Determinants of Interpatient Variability in Treosulfan Pharmacokinetics in AML Patients Undergoing Autologous Stem Cell Transplantation
Source: Int J Mol Sci. 2024 Jul 27;25(15):8215. doi: 10.3390/ijms25158215 (PMC11311427; doi:10.3390/ijms25158215)
Supplement: Supplementary file 1 [file ijms-25-08215-s001.zip › ijms-3114086-supplementary.pdf]

Supplementary Materials

Determinants of Interpatient Variability in Treosulfan Pharmacokinetics in AML Patients Undergoing Autologous Stem Cell Transplantation

Selin G. Ayçiçek [1], Dilara Akhoundova [1], Ulrike Bacher [2], Michael Hayoz [3,4], Yolanda Aebi [3,4], Carlo R. Largiadèr [3,4] and Thomas Pabst [1,*]

[1] Department of Medical Oncology, Inselspital, University of Bern, CH-3010 Bern, Switzerland
[2] Department of Hematology, Inselspital, University of Bern, CH-3010 Bern, Switzerland
[3] Center of Laboratory Medicine (ZLM), Inselspital, University of Bern, CH-3010 Bern, Switzerland
[4] Department of Clinical Chemistry, Inselspital, University of Bern, CH-3010 Bern, Switzerland
[*] Correspondence: thomas.pabst@insel.ch

Table S1. Malnutrition and parenteral nutrition after high-dose chemotherapy followed by autologous stem cell transplantation.

Characteristics	Women ≥ 55 y (*n* = 8)	Men ≥ 55 y (*n* = 16)	Women < 55y (*n* = 14)	Men < 55y (*n* = 17)	All (*n* = 55)	*p*-value
Malnutrition, *n* (%)	4 (50)	10 (59)[A]	12 (86)	10 (59)	36 (66)	0.2719
Malnutrition, NRS						
1	0	1 (6)	1 (7)	4 (24)	6 (11)	0.3561
2	0	1 (6)	0	0	1 (2)	0.6909
3	0	0	2 (14)	4 (24)	6 (11)	0.1135
4	2 (25)	5 (29)	3 (21)	1 (2)	11 (20)	0.2687
5	1 (13)	1 (6)	6 (43)	1 (2)	9 (16)	0.0290
6	1 (13)	2 (12)	0	0	3 (6)	0.1778
TPN needed, *n* (%)	4 (50)	11 (65)	9 (64)	8 (47)	32 (58)	0.5530
Median time under TPN, days (range)	11 (3–15)	9 (3–53)	9 (6–22)	8 (2–12)	9 (2–53)	0.5296

Abbreviations: NRS, nutrition risk screening; TPN, total parenteral nutrition. A NRS of one patient was not defined.

Table S2. Infectious complications.

Characteristics	Women ≥ 55y (*n* = 8)	Men ≥ 55y (*n* = 16)	Women < 55y (*n* = 14)	Men < 55y (*n* = 17)	All (*n* = 55)	*p*-value
Febrile episode	7 (88)	16 (100)	13 (93)	17 (100)	53 (96)	0.1556
Viral infection	0	1 (6)	2 (14)	3 (18)	6 (11)	0.6248
Bacterial infection	5 (63)	6 (38)	5 (36)	11 (65)	27 (49)	0.2588
Fungal infection	1 (13)	0	1 (7)	1 (6)	3 (6)	0.6112

(a)

(b)

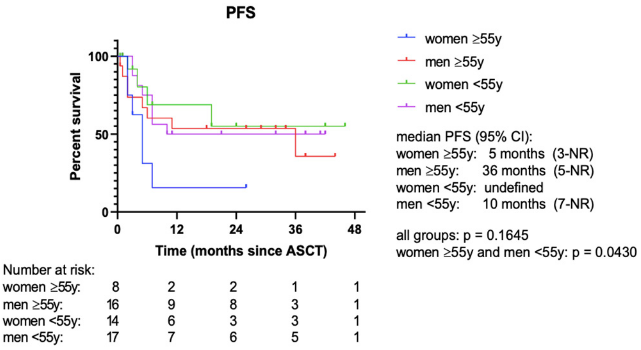

(c)

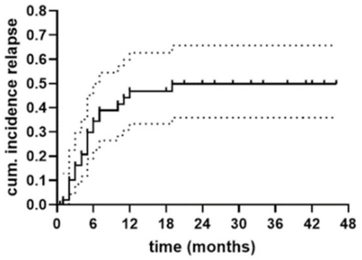

Figure S1. (a) Gender and age-related differences regarding overall survival **(b)** and progression-free survival**. (c)** Cumulative incidence of post-transplant relapse.

(a)

(b) (c)

Figure S2. (a) Treosulfan plasma concentrations within the entire patient cohort. **(b)** Median treosulfan plasma concentrations for all patients. **(c)** Median AUC of treosulfan for all patients.
